# Changes in the prevalence of hepatitis B and C viral infections in Sindh province, Pakistan: Findings from two sero-surveys in 2007 and 2019

**DOI:** 10.1111/jvh.13986

**Published:** 2024-07-26

**Authors:** Tesfa Sewunet Alamneh, Josephine G. Walker, Aaron G. Lim, Ejaz Alam, Saeed Hamid, Graham R. Foster, Naheed Choudhry, M. Azim Ansari, Huma Qureshi, Peter Vickerman

**Affiliations:** 1 Population Health Sciences, Bristol Medical School, University of Bristol, Bristol, UK; 2 Department of Epidemiology and Biostatistics, Institute of Public Health, College of Medicine and Health Sciences, University of Gondar, Gondar, Ethiopia; 3 Pakistan Health Research Council Research Centre, Karachi, Pakistan; 4 Aga Khan University, Karachi, Pakistan; 5 Queen Mary University of London, London, UK; 6 Nuffield Department of Medicine, University of Oxford, Oxford, UK; 7 Doctor Plaza, Karachi, Pakistan

**Keywords:** hepatitis B, hepatitis C, Pakistan

## Abstract

Pakistan harbours a large burden of hepatitis B virus (HBV) and hepatitis C virus (HCV) infection. We utilised repeat sero-surveys to assess progress achieved towards hepatitis elimination in Pakistan. Multilevel logistic regression evaluated the change in HBV infection (HBV surface antigen (HBsAg)-positive) prevalence and HCV exposure (HCV antibody (HCV-Ab)-positive) prevalence between two sero-surveys from 2007 and 2019 for Sindh province and associated risk factors. Adjusted odds ratios (aORs) were estimated and population-attributable fractions (PAF) for modifiable risk factors for HCV exposure. The 2007 and 2019 surveys included 8855 and 6672 individuals. HBsAg prevalence decreased from 2.6% (95% confidence intervals (95% CI): 2.2–2.9) in 2007 to 1.1% (95% CI: 0.8–1.3) in 2019, while HCV-Ab prevalence increased from 5.1% (95% CI: 4.6%–5.5%) to 6.2% (95% CI: 5.6%–6.8%). The age and gender-adjusted HBsAg prevalence decreased by 80% (aOR = 0.2, 95% CI: 0.1–0.4) among children and 60% (aOR = 0.4, 95% CI: 0.3–0.6) among adults over 2007–2019, while HCV-Ab prevalence decreased by 60% (aOR = 0.4, 95%CI:0.2–0.7) in children and increased by 40% (aOR = 1.4, 95% CI: 1.2–1.7) in adults. HCV-Ab prevalence was lower in adults with secondary (aOR = 0.6, 95% CI: 0.5–0.8) and higher (aOR = 0.5, 95%CI:0.3–0.8) education compared to illiterates and higher among adults reporting blood transfusion (aOR = 1.7, 95% CI: 1.2–2.4), family history of hepatitis (aOR = 2.5, 95% CI: 1.9–3.3), past year medical injection (aOR = 2.1, 95% CI: 1.6–2.7), being tattooed (aOR = 1.4, 95% CI: 1.0–1.9) and shaved by traditional barber (aOR = 1.2, 95% CI: 1.0–1.5). Modifiable risk factors accounted for 45% of HCV exposure, with medical injection(s) accounting for 38% (95%CI,25.7–48.4%). Overall HCV has increased over 2007–2019 in Sindh province, while HBV prevalence has decreased. Medical injections should be an important focus of prevention activities.

## 1 Background

Hepatitis B virus (HBV) and hepatitis C virus (HCV) result in bloodborne infections that are associated with increased mortality worldwide.^1^ Individuals infected with HBV and HCV can develop liver cirrhosis, which can lead to hepatocellular carcinoma.^2,3^

Approximately 296 million people were infected with chronic HBV globally and 58 million were infected with HCV in 2019, with these infections resulting in 1.1 million deaths each year.^4^ The burden of HBV and HCV is skewed towards low- and middle-income countries (LMICs), where inadequate resources are available for testing and treatment and infection prevention and control in healthcare settings can be inadequate.^5^ Pakistan ranks among the top four countries globally in terms of viral hepatitis burden.^6^ A nationwide serosurvey from 2007 revealed that HBV and HCV infections were highly endemic in Pakistan.^7^ The national prevalence of HBV infection (HBV surface antigen (HBsAg)-positive) was estimated to be 2.5%, while 4.8% had HCV exposure (HCV antibody (HCV-Ab)-positive), with Sindh province having comparable prevalences (HBsAg 2.5%; HCV-Ab 5%).^7^

The introduction of direct-acting antiviral (DAA) treatments for HCV paired with the widespread availability of vaccination (including birth-dose) for HBV and other prevention interventions for both HCV and HBV (e.g. safety of blood products and safe injections) has led the World Health Organization (WHO) to develop a global health sector strategy for eliminating viral hepatitis as a public health challenge.^8^ This strategy includes targets to reduce the incidence of HCV and HBV infection and their resulting mortality by 2030.^4,9^ This has resulted in many countries moving to increase levels of HBV vaccination among infants, improve injection safety and harm reduction services for people who inject drugs (PWID) and scale up HCV testing and DAA treatment uptake.^10^ For planning interventions to achieve HCV and HBV elimination, it is important to have a good understanding of the epidemiology of these infections, especially in high-prevalence settings such as Pakistan. Sindh conducted a sero-survey in 2019, which estimated the HBsAg prevalence to be 1.1% and HCV-Ab prevalence to be 6.2%. Although these prevalence estimates suggest that the burden of HBV may have decreased in Sindh Province since 2007 while the burden of HCV may have increased, these crude comparisons do not account for any differences between the two survey samples.

To guide ongoing elimination initiatives, we evaluate how the prevalence of HBV infection and HCV exposure has changed in Sindh province between 2007 and 2019. To determine which targeted prevention and screening strategies may be most effective, we assess what risk factors and population characteristics are associated with heightened HBV infection and HCV exposure and determine the population-attributable fractions (PAFs) for all modifiable risk factors associated with HCV exposure.

## 2 Materials and Methods

This analysis utilised two household sero-survey datasets. The first was a nationwide household sero-survey undertaken in 2007.^7^ The survey was carried out in all four provinces of Pakistan, including adults and children from urban and rural areas, to generate representative national data on the prevalence of HBV infection (HBsAg) and exposure to HCV infection (HCV-Ab). The survey collected data on demographic characteristics and risk factors for HBV and HCV from all participants. This included ever history of blood transfusions, invasive dental procedures, injection drug use (IDU), hospitalisation and therapeutic injection in the last year. A two-stage stratified sampling design was employed based on the Federal Bureau of Statistics framework of the 2004 population census update for urban areas and 1998 census data for rural areas since there was no comparable update. Around 7000 households were sampled from 350 primary sampling units (PSUs), 138 from urban and 212 from rural areas. The PSUs were proportionally allocated to the provinces. Of the sampled households, 1560 were from Sindh province. Written consent was obtained from each sampled household. The average size of the sampled households was 6.5 individuals, with every member included. The data-collecting teams asked the local statistical office for replacement households when family members refused to participate or were not present. For all survey participants, both HBsAg and HCV-Ab testing was performed on whole-blood samples using enzyme-linked immunosorbent assay (ELISA).

Similarly, a two-stage stratified sampling design was applied in the second sero-survey of Sindh province in 2019 including all 29 districts. The province was stratified into urban and rural areas as defined in the 2017 census and each city was divided into enumeration blocks by the population census, each including 200–250 households. The enumeration blocks and villages were taken as PSUs. A total of 1160 households were included in the survey, with 40 households being sampled from each district and an average of five individuals included per household. After obtaining written consent from adults 18 years or older and assent for children, data were collected from each available adult in the sampled household, with a separate questionnaire being used to collect information for children from their parents/caregivers. For adults, self-reported data were obtained on similar things as the national survey, but also previous testing and treatment history for HBV, HCV and HIV. HBsAg and HCV-Ab testing was carried out from finger prick samples on 6672 individuals using WHO-prequalified rapid tests (SD Bioline).

Data for Sindh province were extracted from the 2007 national survey and pooled with the data from the 2019 survey after variables were made consistent across the two surveys (Supplementary material—Data S1). Because some districts were split in the 2019 survey, these districts were merged to make it comparable to the 2007 survey. We excluded individuals aged >100 years. Potential risk factors for HBV and HCV (i.e. history of blood transfusions, tattoos or acupuncture, invasive dental procedures, hospitalisation and therapeutic injection) were not collected for children during the 2019 survey, hence in the regression analysis we only determined the risk factors for participants who were adults, defined as being ≥18 years.

### 2.1 Outcome variable

In both surveys, participants were tested for HBsAg and HCV-Ab, with individuals reactive to HBsAg and HCV-Ab testing being considered as individuals infected with HBV and exposed to HCV, respectively.

### 2.2 Demographic and potential risk factors

We extracted demographic characteristics such as gender, age and literacy status. Risk factors including having ever been shaved by a traditional barber, ever undertaken injectable drug use (IDU) (type not specified), having ever been tattooed or received acupuncture, having a therapeutic injection in the last year, having ever had a blood transfusion, being admitted to hospital in the last year and ever having invasive dental treatment were extracted for adults, while history of HBV or HCV in a family member and vaccination for HBV infection were extracted for all participants.

### 2.3 Statistical analysis

STATA 17 was used to analyse the data. We assessed the change in the overall, children and adult prevalence of HBV infection and HCV exposure in Sindh province from 2007 to 2019. We estimated the prevalence of HBV infection and HCV exposure for different demographic characteristics, as well as by different risk factors for HBV and HCV infection across the two surveys. Because of the hierarchical nature of the data for both surveys, with individuals being grouped within households and households being nested within districts, we included clustering by district and household in multilevel binary logistic regression models. First, to evaluate whether the observed changes in prevalence were due to demographic differences in the surveys, regression models estimated the age and gender-adjusted change in HBV or HCV prevalence among either children or adults across the surveys. Following this, additional regression models among adults evaluated the change in prevalence across the surveys while also accounting for potential risk factors for HBV infection and HCV exposure and differences in the prevalence of these risk factors across the rounds. These regression models also assessed the association of these risk factors with the prevalence of HBV infection or HCV exposure among adults. Both bivariable and multivariable analyses were performed in each regression model. In the second set of models (regression model from the pooled dataset), variables with a *p*-value<.2 in the bivariable analysis were included in the multivariable analysis. Finally, separate regression models for each survey round were also undertaken to assess whether the same factors were associated with HBV infection and HCV exposure as for the pooled surveys (Tables S3 and S4). To compare the estimates from the pooled dataset with each survey round, the same set of variables was used in the multivariable regression as in the pooled analysis, regardless of the *p*-value. We reported the unadjusted Odds ratios (ORs) and adjusted odds ratios (aORs) with 95% confidence intervals (CIs).

Lastly, PAFs were calculated using the punaf package in STATA to estimate the proportion of the HCV-Ab prevalence among adults that could be attributed to different modifiable risk factors. We only determined the PAF for modifiable risk factors that had strong associations (*p*-value<.05) in our multivariable regression analysis. PAFs were not calculated for HBV because we had insufficient power to determine clear associations for modifiable risk factors in our multivariable regression analysis.

## 3 Results

This analysis included 8855 and 6672 individuals with available HBV and HCV testing results from the 2007 and 2019 surveys. The median age of the participants was 20 years (interquartile range (IQR):10–35) in the 2007 survey and 20 years (IQR: 9–37) in the 2019 survey. Nearly half of the adults (≥18) in both surveys (48.8% in 2007 and 50.1% in 2019) were illiterate. Approximately three-quarters of adults in both surveys (77.8% in 2007 and 71.5% in 2019) reported receiving therapeutic injections in the last year, while 13.8% in the 2007 survey and 21.6% in the 2019 survey were admitted to hospital in the last year. The prevalence of having a history of HBV or HCV infection in the family increased from 4.3% to 11.6%, the prevalence of ever having a blood transfusion among adults increased from 3.1% to 7.9% and the prevalence of ever having invasive dental procedures increased from 6.1% to 12.4%. Few adults (0.4% in 2007 vs. 0.9% in 2019) reported ever using injectable drugs (Table 1).

The overall prevalence of HBV infection (HBsAg-positive) decreased from 2.6% (95% CI: 2.2%–2.9%) in 2007 to 1.1% (95% CI: 0.8%–1.3%) in 2019 (*p*-value<.001). The prevalence of HBV infection among children (<18) decreased from 1.9% (95% CI: 1.5%–2.3%) in 2007 to 0.6% (95% CI: 0.3%–0.8%) in 2019 (*p*-value<.001) and similarly decreased from 3.1% (95% CI: 2.6%–3.5%) to 1.4% (95% CI: 1.0%–1.8%) among adults (*p*-value<.001). The overall prevalence of HBV infection varied by district in both surveys, with Khairpur having the highest prevalence in 2007 (8.7%) and Sukkur having the highest prevalence in 2019 (4.7%) (Figure S1A). The prevalence of HBV infection was higher among males (3.3%: 95% CI: 2.8%–3.8%) than females (1.8%; 95% CI: 1.4%–2.2%) in 2007 but not in 2019 (1.3%: 95% CI: 0.9%–1.7% among males versus 0.8%: 95% CI: 0.5%–1.1% among females) (Table 2).

The overall prevalence of HCV exposure (HCV-Ab positive) increased from 5.1% (95% CI: 4.6%–5.5%) in 2007 to 6.2% (95% CI: 5.6%–6.8%) in 2019 (*p*-value = .002). The prevalence of HCV exposure among children decreased from 1.3% (95% CI: 0.9%–1.6%) in 2007 to 0.5% (95% CI: 0.3%–0.8%) in 2019 (*p*-value = .002), while it increased from 7.9% (95% CI: 7.2%–8.7%) to 10.8% (95% CI: 9.8%–11.7%) among adults (*p*-value<.001). The prevalence of HCV exposure varied by district across the surveys, with some being hyper-endemic (>10%) and some having lower prevalence (<4%). The prevalence was highest in Khairpur (12.7%) in 2007 and highest in Sanghar (14.5%) in 2019 (Figure S1B). The prevalence also varied with age, being higher in older individuals than younger individuals up until the 50–59 age group (13.1%, 95% CI: 10.5%–16.1% in 2007). HCV exposure prevalence among illiterate adults increased from 9.3% in 2007 to 13.4% in 2019 (Table 3).

### 3.1 Determinants of HBV infection and HCV exposure among adults

The age- and gender-adjusted regression model for HBV showed that the prevalence of HBV infection decreased by 80% among children (aOR = 0.2, 95% CI: 0.1–0.4) over the survey rounds and 60% (aOR = 0.4, 95% CI: 0.3–0.6) among adults (Table S1). When adjusted for all potential covariates, the alternative multivariable regression model for HBV among adults highlighted that being male, having a family history of hepatitis, being in an older age group and earlier survey year were all positively associated with HBV infection (Table 4).

The age- and gender-adjusted regression model for HCV estimated that the prevalence of HCV exposure decreased by 60% among children (aOR = 0.4, 95% CI: 0.2–0.7) but increased by 40% (aOR = 1.4, 95% CI: 1.2–1.7) among adults across the survey rounds (Table S2). When adjusted for all potential covariates, the alternative regression model estimated a smaller odds ratio for the increase in prevalence of HCV exposure among adults over 2007–2019 (aOR = 1.2, 95% CI: 1.0–1.6). Adults from older age groups had a higher prevalence of HCV exposure, but there was no difference in the prevalence of HCV exposure by gender. In comparison to participants who had never attended school, participants with secondary education had 40% lower odds of HCV exposure (aOR = 0.6, 95% CI: 0.5–0.8), while those with higher education had 50% lower odds (aOR = 0.5, 95% CI: 0.3–0.8). There was a greater prevalence of HCV exposure among study participants who had ever had a blood transfusion (aOR = 1.7, 95% CI: 1.2–2.4), ever been tattooed (aOR = 1.4, 95% CI: 1.0–1.9), ever shaved by a traditional barber (aOR = 1.2, 95% CI: 1.0–1.5), or received therapeutic injection(s) in the last year (aOR = 2.1, 95% CI: 1.6–2.7) compared to those that had not. Those with a history of hepatitis in their families had 2.5 times (aOR = 2.5, 95% CI: 1.9–3.3) higher prevalence of HCV exposure than participants without a family history (Table 5).

### 3.2 Population attributable fraction of modifiable risk factors to HCV exposure prevalence among adults

Overall, approximately 45.5% of prevalent HCV exposures were attributable to modifiable risk factors among adults. Receiving therapeutic injections in the last year accounted for 38.1% (95% CI: 25.7%–48.4%) of HCV exposure, while ever having a blood transfusion or being shaved by a traditional barber was responsible for 3.0% (95% CI: 0.9%–5.0%) and 4.4% (95% CI: 0.2%–8.4%) of HCV exposure, respectively (Table 6). Despite this strong association between receiving therapeutic injections and HCV exposure, we found no correlation between the prevalence of therapeutic injection and the prevalence of HCV exposure across districts (correlation coefficient (*r*) = 0.42, *p*-value = .11 in 2007 and *r* = −0.22, *p*-value = .41 in 2019).

## 4 Discussion

This study examined changes in the prevalence of chronic HBV infection and HCV exposure in Sindh province, Pakistan, between 2007 and 2019. After adjusting for age and gender, we found that the prevalence of chronic HBV infection decreased considerably among children (by 80%) and adults (by 60%) over this period, while the prevalence of HCV exposure also decreased in children (by 60%) but increased in adults (by 40%). The prevalence of HBV infection and HCV exposure varied greatly across districts and were generally higher for older ages. The prevalence of HBV infection, but not HCV, was higher in males with both being higher among people who had never attended school or that had a family history of hepatitis. HCV exposure was also heightened among adults with various risk factors, such as ever having been shaved by a traditional barber, ever having received a blood transfusion or tattoo and receiving therapeutic injection(s) in the last year. Of these, receiving therapeutic injection(s) was estimated to be the most important behaviour associated with being exposed to HCV due to its high prevalence (>70%), with over a third of infections being attributable to this risky behaviour.

### 4.1 Strengths and limitations

This is the first study that we know of that has utilised two surveys to assess trends in HBV infection and HCV exposure in Pakistan. Both surveys involved large representative samples from all districts in Sindh, increasing our findings’ reliability and generalisability. In considering whether HBV and HCV prevalence changed over the rounds, we adjusted for demographic factors that may have differed between the surveys, so increasing confidence in our results.

Limitations include not being able to compare levels of active HCV infection between surveys because HCV-RNA testing was not done in the 2007 survey. Some of the increase in HCV seroprevalence among adults may be due to increased life expectancy in those who have been treated, although this is only likely to affect older participants as death from HCV in young people is uncommon. In addition, different diagnostic tests used in the surveys might impact the change in HBV prevalence, with SD Bioline being used in the 2019 survey and ELISA test used in 2007. Although SD Bioline is pre-qualified by WHO for HBV screening, previous studies suggest that it may have lower sensitivity than ELISA.^11–13^ However, this does not seem to be the case with whole blood samples as were used in these surveys. The sampling unit for both surveys was households. This may mean the surveys underestimate the prevalence of HBV infection and HCV exposure because they may under-sample high-risk populations such as people who are homeless, PWIDs, migrants and prisoners, as has been suggested in other settings.^14^

There were also limitations in the survey tools. The surveys did not use the same measures for some risk factors, hindering our comparisons. For instance, we could only look at a history of therapeutic injection/s due to a lack of comparable measures to evaluate how the frequency of therapeutic injections is associated with the risk of HCV exposure.^15^ The periods for risk factors also varied, with some variables asking about ever exposure and others asking about exposure in the last year. Risk factors were not collected for children. This meant we could only consider adults in the regression analyses to identify associated risk factors. Households that refused to participate in the 2007 survey were replaced. Although this is sometimes seen as a weakness, we do not feel this is the case in our study because only a low proportion (4%) were replaced and they were chosen randomly using the same sampling frame.^16,17^ Lastly, since most data were collected through interviews, it is possible that social desirability bias affected the reporting of behavioural variables, especially ever having injected drugs. However, even with the likely under-reporting of this risk behaviour, a relatively high and increasing proportion of study participants reported usage of injectable drugs when compared to other settings, with the estimated prevalence of IDU from 2019 (0.9%) being three times the global average (0.29%).^18^ This agrees with other evidence suggesting the number of PWIDs has increased in Pakistan.^19^ However, the HCV prevalence was unexpectedly low in this population, with IDU not being associated with heightened HCV prevalence. Although this could be due to the small number of people (*n* = 49) reporting this risk behaviour, the lack of association may also suggest that some people misunderstood the question, possibly answering yes if they injected medications.

### 4.2 Comparison with other studies

Our findings are consistent with what has been observed in Punjab, Pakistan, with the 2018 Punjab sero-survey also observing a decrease in overall HBV infection prevalence compared to the 2007 national survey, while HCV exposure also decreased in children and increased in adults.^20,21^ Our findings of an increase in HCV prevalence are consistent with previous HCV modelling by our team,^22^ which predicts an increasing prevalence of HCV infection over this period. However, our findings differ from another modelling study for Pakistan which projects a slow decline in HCV infection.^23^ In line with research from other LMICs,^24–26^ including Pakistan,^27^ we found that males had a higher prevalence of HBV infection than females. In contrast, a study in Nawabshah, Sindh province, found no gender differences in HBV infection prevalence in the general population.^28^ There is not a clear explanation for the gender difference in HBV infection, although studies from Pakistan suggest the use of barbers may be a possible explanation.^29,30^ In agreement with previous studies, our study found that healthcare exposures such as therapeutic injections and blood transfusions, as well as behavioural factors, a family history of hepatitis and socio-demographic factors, are important risk factors for HCV exposure.^15,28,29,31–34^ However, we found a much larger PAF for therapeutic injections (38%) in Sindh than was previously estimated for healthcare exposures from the 2007 national survey (13%).^15^ This seems to be due to a much higher prevalence of HCV exposure among people having therapeutic injections in Sindh compared to what is seen nationally in the 2007 survey.

## 5 Conclusion and Implications

The prevalence of HBV infection is decreasing both in children and adults in Sindh province. The decrease in children could be due to the scale-up of HBV vaccination among infants in Pakistan.^35^ However, the reason for the decrease in prevalence in adults is more uncertain. In Pakistan, only 29% of HBV cases were diagnosed and only 2% of those eligible were receiving treatment in 2020,^36^ suggesting that the country’s treatment services are unlikely to have decreased the infection prevalence. Sindh has a catchup vaccination programme, which may partly account for some of the observed decrease in HBV prevalence in adults; however, this alone cannot explain the large observed change because levels of vaccination in adults are low (3.3%). Otherwise, the decrease could be due to HBV-related death and death due to other chronic conditions and co-morbidities which may co-occur in individuals with HBV. A recent study examining the global, regional and national burden of HBV found that the number of HBV-associated deaths in Pakistan increased by 68% between 1990 and 2019.^37^ Also, evidence suggests the prevalence of non-communicable diseases is very high in Pakistan.^38,39^ Lastly, we observed a greater decrease in HBV prevalence among adults at older ages than in younger adults (OR decreases with age), which potentially aligns with our hypothesis that the decrease could be due to elevated mortality. Overall, the observed decrease in HBV prevalence suggests Sindh may be progressing towards achieving HBV elimination, advocating that the strategies outlined in the national hepatitis strategic framework for Pakistan should be continued and strengthened to decrease incidence further.^40,41^ These strategies include administering infant HBV vaccination, ensuring blood and injection safety and improving harm reduction services for PWIDs.

HCV exposure in Sindh has increased overall, while it has decreased in children and increased in adults. Although the reason for the decrease in prevalence among children remains unclear, it provides hope that the epidemic might be turning. It also emphasises the need for greater surveillance in children. Interestingly, the increase in prevalence of HCV exposure among adults over the survey rounds becomes marginal after adjustment for potential covariates. This could be because the increase in HCV exposure resulted from an increase in risk factors which also occurred between the surveys, hence adjusting for these risk factors attenuated the association between the survey year and HCV exposure.

HCV is typically acquired through the contamination of blood or blood products.^42^ Although blood transfusion used to be an important risk factor for acquiring HCV, this is thought to have diminished since Pakistan initiated mandatory blood screening for transfusion transmittable infections.^43^ However, despite this, HCV transmission is still high, with our analysis suggesting that in adults this is mainly driven by continued blood contamination from unsafe therapeutic injections. These occur at a high frequency in Pakistan compared to other countries^44^ and frequently occur in private medical settings by unqualified practitioners with sub-standard infection control practices.^45^ The common practice of community barbering, the increased popularity of tattooing or acupuncture and increases in IDU in Pakistan may also be adding to this high risk. To reduce the risk of HCV exposure and future disease morbidity, it is crucial that Sindh and Pakistan deal with this increasing burden of HCV. Although an increase in the prevalence of a family history of hepatitis across the surveys might point to an increase in screening and people’s awareness of their status, there is still an urgent need to scale-up HCV screening and treatment to prevent current infections progressing to liver disease and to reduce ongoing transmission in Sindh and elsewhere in Pakistan. Future prevention initiatives also need to have a strong focus on formal and informal healthcare exposures, especially unsafe medical injections, to stem the province’s main source of HCV transmission. This should include increasing awareness about the risks of unsafe medical injections and the importance of using new syringes, as well as encouraging other forms of drug delivery to reduce the frequency of injections while improving their safety. Without combining these and other strategies, Pakistan will not be able to control their expanding HCV epidemic and will not progress to achieving the HCV elimination targets set by WHO.

## Supplementary Material

Data S1.

Figure S1.

Figure S2.

## Figures and Tables

**Table 1:** Background characteristics of the study participants in the 2007 and 2019 sero-survey in Sindh, Pakistan.

		2007 survey			2019 survey	
Variable	Category	Frequency	Percent		Frequency	Percent
District	Badin	356	4.0		219	3.3
	Dadu	505	5.7		493	7.4
	Ghotki	202	2.3		260	3.9
	Hyderabad	1082	12.2		842	12.6
	Jacobabad	393	4.4		434	6.5
	Karachi	2185	24.7		1331	19.6
	Khairpur	474	5.4		265	4.0
	Larkana	420	4.7		518	7.8
	Mirpur Khas	566	6.4		470	7.0
	Nawab Shah	356	4.0		236	3.5
	Nosheroferoz	224	2.5		255	3.8
	Sanghar	643	7.3		242	3.6
	Shikarpur	300	3.4		227	3.4
	Sukkur	456	5.2		173	2.6
	Tharparkar	305	3.4		237	3.6
	Thatta	388	4.4		470	7.0
Gender	Female	4218	47.6		3504	52.5
	Male	4637	52.4		3160	47.4
	Transgender	–	–		8	0.1
Participants group	Child	3826	43.2		2988	44.78
	Adult	5029	56.8		3684	55.22
Age group	<5	827	9.34		633	9.49
	5–9	1163	13.13		1126	16.88
	10–17	1836	20.73		1229	18.42
	18–29	2067	23.34		1283	19.23
	30–39	1087	12.28		907	13.59
	40–49	804	9.08		634	9.50
	50–59	580	6.55		409	6.13
	60–69	302	3.41		310	4.65
	≥70	189	2.13		141	2.11
Reported family hepatitis history		377/8855	4.3		768/6637	11.6
HBV vaccination	No	8465	95.6		5305	80.3
	Yes	390	4.4		1305	19.7
Variables reported for adult participants only						
Educational level	Illiterate	2452	48.8		1845	50.1
	Primary	929	18.5		622	16.9
	Secondary	1167	23.2		571	15.5
	Tertiary	380	7.6		180	4.5
	Other	101	2.0		–	–
	Missing	–	–		466	12.7
Reported tattoo/acupuncture^b^		56/5029			500/3684	13.6
Reported traditional barber^b^		1168/5029	23.2		991/3521	28.2
Reported blood transfusion^b^		156/5029	3.1		290/3651	7.9
Reported hospitalisation^a^		696/5029	13.8		793/3665	21.6
Reported invasive dental procedure^b^		308/5029	6.1		455/3672	12.4
Reported IDU^b^		18/5029	0.4		31/3664	0.9
Reported therapeutic injection^a^		3913/5029	77.8		2623/3669	71.5

a Reported history in the last year.

b Reported ever history.

**Table 2:** Prevalence of active hepatitis B infection (HBsAg-positive) by potential risk factors in Sindh province, Pakistan, between 2007 and 2019.

		HBsAg+ 2007				HBsAg+ 2019		
Variable	Category	Frequency	Percent	95% CI		Frequency	Percent	95% CI
Gender	Female	74	1.8	1.4–2.2		28	0.8	0.5–1.1
	Male	152	3.3	2.8–3.8		42	1.3	0.9–1.7
	Transgender	–	–	–		0	0	–
Participant group	Child (<18)	72	1.9	1.5–23		18	0.6	0.3–0.8
	Adult (≥18)	154	3.1	2.6–3.5		52	1.4	1.0–1.8
Age	<5	10	1.2	0.6–2.2		2	0.3	0.03–1.1
	5–9	22	1.9	1.2–2.9		8	0.7	0.3–1.4
	10–17	40	2.2	1.6–3.0		8	0.8	0.4–1.4
	18–29	46	2.2	1.6–3.0		27	2.1	1.4–3.1
	30–39	35	3.2	2.3–4.4		11	1.2	0.6–2.2
	40–49	20	2.5	1.5–3.8		9	1.4	0.7–2.7
	50–59	32	5.5	3.8–7.7		3	0.7	0.2–2.1
	60–69	14	4.6	2.6–7.7		0	0	–
	≥70	7	3.7	1.5–7.5		2	1.4	0.2–2.1
Family hepatitis history	No	215	2.5	2.2–2.9		52	0.9	0.7–1.2
	Yes	11	2.9	1.5–5.2		17	2.2	1.3–3.5
HBV vaccination	No	225	2.7	2.3–3.0		63	1.2	0.9–1.5
	Yes	1	0.3	0.01–1.4		6	0.5	0.2–1.0
Educational level^c^	Illiterate	81	3.3	2.6–4.1		28	1.5	1.0–2.2
	Primary	38	4.1	2.9–5.5		7	1.1	0.5–2.3
	Secondary	27	2.3	1.5–3.3		10	1.5	0.8–3.2
	Tertiary	6	1.6	0.6–3.4		5	2.8	0.9–6.4
	Missing	–	–	–		2	0.4	0.1–1.5
	Other	2	2	0.2–6.9		–	–	–
Tattoo/acupuncture^b,c^	No	153	3.1	2.6–3.6		44	1.4	1.0–1.8
	Yes	1	1.8	0.1–9.6		8	1.6	0.5–2.7
Traditional barber^b,c^	No	100	2.6	2.1–3.1		27	1.1	0.7–1.5
	Yes	54	4.6	3.5–6.0		24	2.4	1.5–3.2
Blood transfusion^b,c^	No	151	3.1	2.6–3.6		49	1.5	1.1–1.9
	Yes	3	1.9	0.4–5.5		3	1.0	0.2–3.0
Hospitalisation history^a,c^	No	140	3.2	2.7–3.8		39	1.4	1.0–1.9
	Yes	14	2.0	1.1–3.4		13	1.6	0.9–2.8
Invasive dental procedure^b,c^	No	149	3.2	3.0–4.1		47	1.5	1.1–1.9
	Yes	5	1.6	0.5–3.8		5	1.1	0.4–2.6
Injectable drug use^b,c^	No	154	3.1	2.6–3.6		51	1.4	1.1–1.8
	Yes	0	0	–		1	3.2	0.1–16.7
Therapeutic injection^a,c^	No	26	2.3	1.5–3.4		14	1.3	0.7–2.2
	Yes	128	3.1	2.7–3.9		38	1.5	1.0–2.0
Total		226	2.6	2.2–2.9		77	1.1	0.8–1.3

a History in the last year.

b Ever history.

c Among adults only.

**Table 3:** Prevalence of hepatitis C exposure (HCV-Ab-positive) by potential risk factors in Sindh province, Pakistan, between 2007 and 2019.

		HCV-Ab^+^ 2007				HCV-Ab^+^ 2019		
Variable	Category	Frequency	Percent	95% CI		Frequency	Percent	95% CI
Gender	Female	214	5.1	4.4–5.8		230	6.6	5.8–7.4
	Male	234	5.1	4.4–5.7		183	5.8	5.0–6.7
	Transgender	–	–	–		0	0	–
Participant group	Child (<18)	48	1.3	0.9–1.6		16	0.5	0.3–0.8
	Adult (>18)	400	7.9	7.2–8.7		397	10.8	9.8–11.7
Age	<5	8	1.0	0.4–1.9		1	0.2	0.004–0.9
	5–9	8	0.7	0.3–1.4		4	0.4	0.1–0.9
	10–17	32	1.7	1.2–2.5		11	0.9	0.4–1.6
	18–29	93	4.5	3.6–5.5		53	4.1	3.1–5.4
	30–39	102	9.4	7.7–11.3		100	11.0	9.1–13.2
	40–49	90	11.2	9.1-13.4		85	13.4	10.9–16.3
	50–59	76	13.1	10.5–16.1		73	17.9	14.3–21.9
	60–69	27	8.9	6.0–12.7		66	21.3	16.9–26.3
	≥70	12	6.4	3.3–10.8		20	14.2	8.9–21.1
Family hepatitis history	No	403	4.8	4.3–5.2		311	5.3	4.7–5.9
	Yes	45	11.9	8.8–15.6		100	13.0	10.7–15.6
HBV vaccination	No	444	5.3	4.8–5.7		376	7.1	6.4–7.8
	Yes	4	1.03	0.3–2.6		29	2.2	1.5–3.2
Educational level^c^	Illiterate	227	9.3	8.2–10.5		248	13.4	11.9–15.1
	Primary	89	9.6	7.8–11.7		60	9.7	7.4–12.2
	Secondary	61	5.2	4.0–6.7		29	5.1	3.4–7.2
	Tertiary	16	4.2	2.4–6.7		10	5.6	2.7–10.0
	Other	7	6.9	2.8–13.8		–	–	–
	Unknown	–	–	–		50	10.7	8.1–13.9
Tattoo/acupuncture^b,c^	No	394	7.9	7.2–8.7		325	10.2	9.2–11.3
	Yes	6	10.7	4.0–21.9		72	14.4	11.4–17.8
Traditional barber^b,c^	No	288	7.5	6.7–8.3		266	10.5	9.4–11.8
	Yes	112	9.6	9.5–13.6		113	11.4	9.5–13.6
Blood transfusion^b,c^	No	366	7.5	6.8–8.3		352	10.5	9.46–11.6
	Yes	34	21.8	15.6–29.1		43	14.8	10.9–19.5
Hospitalisation history^a,c^	No	321	7.4	6.7–8.2		284	9.9	8.8–11.0
	Yes	79	11.4	9.1–14.0		110	13.9	11.4–16.3
Invasive dental procedure^b,c^	No	378	8.01	7.3–8.8		336	10.4	9.4–11.6
	Yes	22	7.1	4.5–10.6		59	13.0	10.0–16.4
Injectable drug use^b,c^	No	398	7.9	7.2–8.7		392	10.8	9.8–11.8
	Yes	2	11.1	1.4–34.7		3	9.9	2.0–25.8
Therapeutic injection^a,c^	No	28	2.5	1.7–3.6		81	7.7	6.2–9.5
	Yes	372	9.5	8.6–10.5		315	12.0	10.8–13.3
Total		448	5.1	4.6–5.5		413	6.2	5.6–6.8

a History in the last year.

b Ever history.

c Among adults only.

**Table 4:** Factors associated with the prevalence of hepatitis B infection (HBsAg-positive) among adults in Sindh province, Pakistan, from 2007 to 2019 sero-surveys.

Variable	Category	OR	95% CI	*p*-value	aOR	95% CI	*p*-value
Gender	Female	1	1		1	1	
	Male	2.2	1.6–2.9	<.01	1.8	1.2–2.7	.002
Age	18–29	1	1		1	1	
	30–39	1.0	0.7–1.5	.96	1.1	0.7–1.6	.65
	40–49	0.9	0.6–1.4	.65	1.0	0.6–2.7	.90
	50–59	1.6	1.1–2.5	.03	1.7	1.1–2.7	.02
	60–69	1.0	0.5–1.8	.93	1.1	0.6–2.0	77
	≥70	1.2	0.5–2.5	.68	1.2	0.6–2.6	.63
Educational level	Illiterates	1	1	–			
	Primary	1.2	0.8–1.8	.31			
	Secondary	0.9	0.6–1.4	.66			
	Higher	0.9	0.5–1.8	.76			
	Other	0.8	0.2–3.4	.72			
Tattoo/acupuncture^b^	No	1	1		1	1	
	Yes	0.6	0.3–1.2	.16	1.0	0.4–2.3	.96
Traditional barber^b^	No	1	1		1	1	
	Yes	1.8	1.3–2.5	<.001	1.3	0.9–1.8	.23
Blood transfusion history^b^	No	1	1		1	1	
	Yes	0.5	0.2–1.2	.09	0.6	0.2–1.6	.29
Hospitalisation history^a^	No	1	1				
	Yes	0.7	0.4–1.4	.26			
Invasive dental procedure^b^	No	1	1		1		
	Yes	0.6	0.3–1.2	.14	0.6	0.3–1.3	.24
Family history of Hepatitis	No	1	1		1	1	
	Yes	1.3	0.8–2.2	.14	1.8	1.1–3.0	.03
HBV vaccination	No	1	1		1	1	
	Yes	0.3	0.8–1.4	.13	0.3	0.1–1.5	.12
Therapeutic injection^a^	No	1	1		1	1	
	Yes	1.4	1.0–2.1	.09	1.3	0.9–1.9	.20
Injection drug use	No	1	1				
	Yes	1.1	0.1–9.1	.91			
Survey year	2007	1	1		1	1	
	2019	0.4	0.3–0.6	<.01	0.4	0.3–0.7	<.001

Abbreviations: aOR, adjusted odds ratio; OR, odds ratio.

a History in the last year.

b Ever history.

**Table 5:** Factors associated with the prevalence of hepatitis C exposure (HCV-Ab-positive) among adults in Sindh province, Pakistan, from 2007 to 2019 sero-surveys.

Variable	Category	OR	95% CI	*p*-value	aOR	95% CI	*p*-value
Gender	Female	1	1	–			
	Male	0.9	0.8–1.1	.23			
Age	18–29	1	1		1	1	
	30–39	2.7	2.1–3.4	<.001	2.5	1.9–3.2	<.001
	40–49	3.5	2.7–4.5	<.001	3.0	2.3–3.9	<.001
	50–59	4.7	3.6–6.3	<.001	4.0	3.0–5.4	<.001
	60–69	4.7	3.4–6.5	<.001	3.7	2.6–5.1	<.001
	>70	2.7	1.7–4.3	<.001	2.2	1.4–3.5	.001
Educational level	Illiterates	1	1		1	1	
	Primary	0.8	0.7–1.0	.09	0.9	0.7–1.2	.63
	Secondary	0.4	0.3–0.6	<.001	0.6	0.5–0.8	.001
	Higher	0.4	0.3–0.6	<.001	0.5	0.3–0.8	.005
	Other	0.6	0.2–1.4	.024	0.8	0.3–2.0	.65
	Unknown	1.1	0.7–1.7	.59	1.1	0.7–1.7	.79
Tattoo/acupuncture^b^	No	1	1		1	1	
	Yes	1.4	1.2–2.2	.05	1.4	1.0–1.9	.08
Traditional barber^b^	No	1	1		1	1	
	Yes	1.2	1.0–1.4	.06	1.2	1.0–1.5	.03
Blood transfusion history^b^	No	1	1		1	1	
	Yes	2.5	1.9–3.4	<.001	1.7	1.2–2.4	.002
Hospitalisation history^a^	No	1	1		1	1	
	Yes	1.9	1.6–2.4	<.001	1.1	0.8–1.5	.77
Invasive dental procedure^b^	No	1	1		1	1	
	Yes	1.6	1.2–2.1	.04	0.9	0.7–1.2	.53
Family history of hepatitis^b^	No	1	1		1	1	
	Yes	2.	2.2–3.8	<.001	2.5	1.9–3.3	<.001
HBV vaccination	No	1	1		1	1	
	Yes	1.5	0.9–2.3	.13	1.2	0.7–2.0	.56
Therapeutic injection^a^	No	1	1		1	1	
	Yes	2.3	1.8–3.0	<.001	2.1	1.6–2.7	<.001
Injection drug use	No	1	1	–			
	Yes	1.5	0.5–4.4	.41			
Survey year	2007	1	1		1	1	
	2019	1.5	1.2–1.8	<.001	1.2	1.0–1.6	.08

Abbreviations: aOR, adjusted odds ratio; OR, odds ratio.

a History in the last year.

b Ever history.

**Table 6:** Population attributable fractions (PAFs) of modifiable risk factors for hepatitis C exposure (HCV-Ab-positive) among adults.

Variable	Frequency	Percent	PAF (95% CI)
Therapeutic injection in the last year	6536	75.1	38.1% (25.7%–48.4%)
Ever blood transfusion history	446	5.1	3.0% (0.9%–5.0%)
Ever being shaved by traditional barber	2159	25.3	4.4% (0.2%–8.4%)
Ever had tattoo/acupuncture	556	6.4	1.7% (−0.4%–3.9%)^a^
Total	–	–	45.5%

a 95% CI includes zero.

## Data Availability

The data that support the findings of this study are available from the corresponding author upon reasonable request.

## Abbreviations

95%CI: 95% confidence interval
aORs: adjusted odds ratios
DAA: direct-acting antiviral
ELISA: enzyme-linked immunosorbent assay
HBsAg: HBV surface antigen
HBV: hepatitis B virus
HCV: hepatitis C virus
HCV-Ab: HCV antibody
IDU: injectable drug use
IQR: interquartile range
LMICs: low and middle-income countries
PAF: population-attributable fractions
PSUs: primary sampling units
PWIDs: people who inject drugs
WHO: World Health Organisation.
