# Supplementary material for: Changes in the prevalence of hepatitis B and C viral infections in Sindh province, Pakistan: Findings from two sero-surveys in 2007 and 2019
Source: J Viral Hepat. Author manuscript; Available in PMC 2024 Dec 23. (PMC7617255; doi:10.1111/jvh.13986)
Supplement: Data S1. [file EMS200023-supplement-Data_S1_.docx]

**Changes in the prevalence of hepatitis B and C viral infections in Sindh province, Pakistan: findings from two sero-surveys in 2007 and 2019**

**Tesfa Sewunet Alamneh^1,2^, Josephine Walker^1^, Aaron G Lim^1^, Ejaz Alam^3^, Saeed Hamid^4^, Graham R Foster^5^, Naheed Choudhry^5^, M. Azim Ansari^6^, Huma Qureshi^7^, Peter Vickerman^1^**

## ^1^Population Health Sciences, Bristol Medical School, University of Bristol, Bristol, UK

## ^2^Department of Epidemiology and Biostatistics, Institute of Public Health, College of Medicine and Health Sciences, University of Gondar, Ethiopia

## ^3^Pakistan Health Research Council Research Centre, Karachi, Pakistan

## ^4^Aga Khan University, Karachi, Pakistan,

## ^5^Queen Mary University of London, London, UK

## ^6^Nuffield Department of Medicine, University of Oxford, Oxford, UK

## ^7^Consultant Gastroenterologist, Doctor Plaza, Clifton Karachi

Supplementary materials

This study utilised data from two household sero-surveys conducted in 2007 as part of the Pakistan national hepatitis survey and the Sindh provincial survey in 2019. Data for the Sindh province was extracted from the 2007 National survey and pooled with data from the 2019 survey. Certain variables were not measured consistently across the surveys. For instance, the 2007 survey recorded educational levels (illiterate, less than primary, primary but less, middle, middle but less, intermediate, matric, degree and above, and others), while the 2019 survey reported the number of years of schooling completed. To ensure consistency between the variables, participant educational levels from the 2007 survey were recoded as follows: primary (combining less than primary, primary but less, and middle), secondary (combining middle but less, matric, and intermediate), and tertiary (degree and above); illiterates and others were kept as such^1^. In the 2019 survey, the years of schooling completed were categorized as follows: illiterate (zero years), primary (1 to 8 years), secondary (9 to 12 years), tertiary (13 to 16 years)^1^, and unknown (missing). Additionally, the number of districts was not the same over the two surveys. There were 29 districts in the 2019 survey, namely Badin, Dadu, Ghotki, Hyderabad, Jamshoro, Jacobabad, Karachi Central, Karachi East, Karachi South, Karachi West, Kashmore / Kand, Khairpur, Korangi, Larkana, Malir, Matiari, Mirpur Khas Naushahro Firoz, Qamber Shahdad, Sajawal, Sanghar, Shaheed Benazir, Shikarpur, Sukkur, Tando Allahyar, Tando MKhan, Tharparkar, Thatta and Umer Kot. These districts were merged accordingly into the following 16 districts, which were available in the 2007 survey.

1. Badin: Badin
2. Dadu: Dadu and Jamshoro ^2^
3. Ghotki: Ghotki
4. Hyderabad: Hyderabad, Matiari, Tando Allahyar and Tando M Khan ^3^
5. Jacobabad: Jacobabad and Kashmore / Kand ^4^
6. Karachi: Karachi Central, Karachi East, Karachi South, Karachi West, Korangi and Malir ^5^
7. Khairpur: Khairpur
8. Larkana: Larkana and Qamber Shahdad ^6^
9. Mirpur Khas: Mirpur Khas and Umer Kot
10. Nawab Shah: Shaheed Benazir
11. Naushahro Firoz: Naushahro Firoz
12. Sanghar: Sanghar
13. Shikarpur: Shikarpur
14. Sukkur: Sukkur
15. Tharparkar: Tharparkar
16. Thatta: Thatta and Sajawal ^7^

Supplementary Table 1. The associations of age and gender with chronic hepatitis B infection (HBsAg-positive) in 2007 and 2019 sero-surveys.

| Strata | Variable | Category | OR | 95%CI | P-value | aOR | 95%CI | P-value |
| --- | --- | --- | --- | --- | --- | --- | --- | --- |
| Children (<18) | Age | <5 | 1 | 1 | - | 1 | 1 | - |
|  |  | 5-9 | 1.9 | 0.8-4.4 | 0.12 | 2.0 | 0.9-4.6 | 0.11 |
|  |  | 10-17 | 3.1 | 1.6-6.8 | 0.01 | 2.9 | 1.3-6.7 | 0.01 |
|  | Gender | Female | 1 | 1 | - | 1 | 1 | - |
|  |  | Male | 1.7 | 1.0-2.8 | 0.05 | 1.6 | 1.0-2.8 | 0.08 |
|  | Survey year | 2007 | 1 | 1 | - | 1 | 1 | - |
|  |  | 2019 | 0.2 | 0.1-0.4 | <0.001 | 0.2 | 0.1-0.4 | <0.001 |
| Adults (>=18) | Age | 18-29 | 1 | 1 | - | 1 | 1 | - |
|  |  | 30-39 | 1.0 | 0.7-1.5 | 0.96 | 1.0 | 0.7-1.6 | 0.85 |
|  |  | 40-49 | 0.9 | 0.6-1.4 | 0.65 | 0.9 | 0.6-1.4 | 0.68 |
|  |  | 50-59 | 1.6 | 1.1-2.5 | 0.03 | 1.6 | 1.0-2.5 | 0.04 |
|  |  | 60-69 | 1.0 | 0.5-1.8 | 0.93 | 1.0 | 0.5-1.9 | 0.99 |
|  |  | >70 | 1.2 | 0.5-2.5 | 0.68 | 1.1 | 0.5-2.3 | 0.85 |
|  | Gender | Female | 1 | 1 | - | 1 | 1 | - |
|  |  | Male | 2.2 | 1.6-2.9 | <0.001 | 2.1 | 1.5-2.8 | <0.001 |
|  | Survey year | 2007 | 1 | 1 | - | 1 | 1 | - |
|  |  | 2019 | 0.4 | 0.3-0.6 | <0.001 | 0.4 | 0.3-0.6 | <0.001 |

Supplementary Table 2. The associations of age and gender with hepatitis C exposure (HCV-Ab-positive) in 2007 and 2019 sero-surveys.

| Strata | Variable | Category | OR | 95%CI | P-value | aOR | 95%CI | P-value |
| --- | --- | --- | --- | --- | --- | --- | --- | --- |
| Children (<18) | Age | <5 | 1 | 1 | - | 1 | 1 | - |
|  |  | 5-9 | 0.9 | 0.3-2.1 | 0.73 | 0.9 | 0.3-2.2 | 0.78 |
|  |  | 10-17 | 2.7 | 1.2-5.9 | <0.001 | 2.7 | 1.2-5.9 | 0.016 |
|  | Gender | Female | 1 | 1 | - | 1 | 1 | - |
|  |  | Male | 1.1 | 0.6-1.8 | 0.77 | 1.0 | 0.6-1.8 | 0.93 |
|  | Survey year | 2007 | 1 | 1 | - | 1 | 1 | - |
|  |  | 2019 | 0.4 | 0.2-0.7 | 0.003 | 0.4 | 0.2-0.7 | <0.001 |
| Adults (>=18) | Age | 18-29 | 1 | 1 | - | 1 | 1 | - |
|  |  | 30-39 | 2.7 | 2.1-3.4 | <0.001 | 2.6 | 2.1-3.4 | <0.001 |
|  |  | 40-49 | 3.5 | 2.7-4.5 | <0.001 | 3.4 | 2.7-4.5 | <0.001 |
|  |  | 50-59 | 4.7 | 3.6-6.3 | <0.001 | 4.7 | 3.6-6.3 | <0.001 |
|  |  | 60-69 | 4.7 | 3.4-6.5 | <0.001 | 4.6 | 3.3-6.3 | <0.001 |
|  |  | >70 | 2.7 | 1.7-4.3 | <0.001 | 2.7 | 1.7-4.3 | <0.001 |
|  | Gender | Female | 1 | 1 | - | 1 | 1- |  |
|  |  | Male | 0.9 | 0.8-1.1 | 0.23 | 0.9 | 0.7-1.0 | 0.10 |
|  | Survey year | 2007 | 1 | 1 | - |  | 1 | 1 |
|  |  | 2019 | 1.5 | 1.2-1.8 | <0.001 | 1.4 | 1.2-1.7 | 0.001 |

A separate analysis for each survey round suggested that the likelihood of HBV infection was just associated (positively) with a family history of hepatitis in 2019, and was higher in males and those aged 50-59 compared to 18-29 in the 2007 survey (Supplementary Table 3). Compared to illiterates, those with secondary and higher education levels were less likely to have HCV exposure in 2019, whereas there was no association in 2007. The HCV exposure prevalence was higher in older people in both survey rounds. People with a history of blood transfusions had a higher prevalence of HCV exposure in 2007, but this association was not observed in the 2019 survey. HCV exposure was positively associated with a history of therapeutic injections in the last year in both surveys, although the odds decreased from 2007 (aOR=3.3: 95% CI: 2.1-5.2) to 2019 (aOR=1.6: 95% CI: 1.2-2.2). People reported ever being shaved by a traditional barber had a higher prevalence of HCV exposure in 2019, but not in 2007. HBV vaccination was found to be independently associated with HCV exposure. It was associated negatively in 2007 but positively in 2019 (Supplementary Table 4).

Supplementary Table 3. Factors associated with the prevalence of chronic hepatitis B infection (HBsAg-positive) in 2007 and 2019 sero-surveys.

|  |  | 2007 survey estimates | | | | 2019 survey estimates | | | |
| --- | --- | --- | --- | --- | --- | --- | --- | --- | --- |
| Variable | Category | OR (95%CI) | p-value | aOR (95%CI) | p-value | OR (95%CI) | p-value | aOR (95%CI) | p-value |
| Gender | Female | 1 |  |  |  | 1 |  |  |  |
|  | Male | 2.2 (1.5-3.2) | <0.001 | 2.1 (1.3 -3.2) | 0.001 | 1.8 (1.0-3.2) | 0.05 | 1.3 (0.6-3.2) | 0.514 |
| Age | 18-29 | 1 |  | 1 |  | 1 |  | 1 |  |
|  | 30-39 | 1.3 (0.8-2.2) | 0.228 | 1.4 (0.8-2.2) | 0.224 | 0.6 (0.3-1.2) | 0.141 | 0.7 (0.3-1.40) | 0.279 |
|  | 40-49 | 1.1 (0.6-1.9) | 0.798 | 1.1 (0.6-1.9) | 0.806 | 0.7 (0.3-1.4) | 0.293 | 0.8 (0.3-1.7) | 0.55 |
|  | 50-59 | 2.5 (1.5-4.2) | <0.001 | 2.5 (1.5-4.1) | <0.001 | 0.3 (0.1-1.2) | 0.094 | 0.4 (0.1-1.6) | 0.208 |
|  | 60-69 | 2.0 (1.0-3.7) | 0.042 | 1.9 (1-4.1) | 0.053 | - |  | - |  |
|  | >70 | 1.5 (0.6-3.7) | 0.352 | 1.4 (0.6-3.5) | 0.449 | 0.7 (0.2-3.2) | 0.636 | 0.9 (0.2-4.1) | 0.856 |
| Educational level | illiterates | 1 |  |  |  | 1 |  |  |  |
|  | Primary | 1.4 (0.9-2.2) | 0.135 |  |  | 0.7 (0.3-1.8) | 0.488 |  |  |
|  | Secondary | 0.8 (0.5-1.3) | 0.405 |  |  | 1.2 (0.5-2.6) | 0.678 |  |  |
|  | Higher | 0.6 (0.2-1.5) | 0.286 |  |  | 2.0 (0.6-5.9) | 0.234 |  |  |
|  | other | 0.6 (0.1-2.8) | 0.523 |  |  | - | - |  |  |
|  | Unknown | - | - | - |  | 0.3 (0.04-1.6) | 0.151 |  |  |
| Tattoo/acupuncture‡ | No | 1 | - | 1 |  | 1 |  | 1 |  |
|  | Yes | 0.6 (0.1-4.5) | 0.581 | 0.6 (0.1-4.6) | 0.595 | 0.8 (0.3-2.1) | 0.727 | 0.9 (0.3-2.7) | 0.896 |
| Traditional barber‡ | no | 1 |  | 1 |  | 1 |  | 1 |  |
|  | yes | 1.8 (1.2-2.5) | 0.03 | 1.1 (0.7-1.7) | 0.623 | 2.1 (1.1-3.8) | 0.016 | 1.6 (0.7-3.8) | 0.272 |
| Blood transfusion history‡ | No | 1 |  | 1 |  | 1 |  | 1 |  |
|  | Yes | 0.5 (0.2-1.8) | 0.316 | 0.6 (0.2-2.1) | 0.436 | 0.6 (0.2-2.1) | 0.436 | 0.5 (0.1-2.3) | 0.359 |
| Hospitalization history† | No | 1 |  |  |  | 1 |  |  |  |
|  | Yes | 0.7 (0.7-1.2) | 0.172 |  |  | 1.1 (0.6-2.2) | 0.30 |  |  |
| Invasive dental procedure‡ | No | 1 |  | 1 |  | 1 |  | 1 |  |
|  | Yes | 0.7 (0.3-1.8) | 0.429 | 0.7 (0.3-1.8) | 0.444 |  |  | 0.7 (0.2-2.2) | 0.532 |
| Family history of hepatitis‡ | No | 1 |  | 1 |  | 1 |  | 1 |  |
|  | Yes | 1.2 (0.6-2.5) | 0.644 | 1.2 (0.6-2.6) | 0.561 | 2.7 (1.3-5.8) | 0.01 | 3.2 (1.4-7.0) | 0.005 |
| HBV vaccination | No | 1 |  | 1 |  | 1 |  | 1 |  |
|  | Yes | 0.2 (0.03-1.8) | 0.16 | 0.2 (0.03-1.9) | 0.181 | 0.8 (0.1-6.4) | 0.826 | 0.7 (0.1-5.8) | 0.740 |
| Therapeutic injection† | No | 1 |  | 1 |  | 1 |  | 1 |  |
|  | Yes | 1.3 (0.8-2.1) | 0.311 | 1.3 (0.8-2.2) | 0.310 | 1.2 (0.6-2.4) | 0.552 | 1.6 (0.8-3.3) | 0.217 |
| Injection drug use | No | 1 |  |  |  | 1 |  |  |  |
|  | Yes | - | - |  |  | 2.3 (0.3-19.7) | 0.462 |  |  |

1. †History in the last year
2. ‡Ever history
3. *Among adults only
4. Supplementary Table 4. Factors associated with the prevalence of hepatitis C exposure (HCV-Ab-positive) in 2007 and 2019 sero-surveys.

|  |  | 2007 survey estimates | | | | 2019 survey estimates | | | |
| --- | --- | --- | --- | --- | --- | --- | --- | --- | --- |
| Variable | Category | OR (95%CI) | p-value | aOR (95%CI) | p-value | OR (95%CI) | p-value | aOR (95%CI) | p-value |
| Gender | Female | 1 |  |  |  | 1 |  |  |  |
|  | Male | 0.9(0.7-1.2) | 0.457 |  |  | 0.9 (0.7-1.2) | 0.53 |  |  |
| Age | 18-29 | 1 |  | 1 |  | 1 |  | 1 |  |
|  | 30-39 | 2.4 (1.7-3.3) | <0.001 | 2.1 (1.5-3.0) | <0.001 | 3.2 (2.2-4.6) | <0.001 | 3.1 (2.1-4.7) | <0.001 |
|  | 40-49 | 2.9 (2.1-4.1) | <0.001 | 2.4 (1.7-3.5) | <0.001 | 4.3 (2.8-6.4) | <0.001 | 3.9 (2.5-5.9) | <0.001 |
|  | 50-59 | 3.8 (2.6-5.5) | <0.001 | 3.3 (2.2-4.8) | <0.001 | 6.4 (4.2-9.7) | <0.001 | 5.3 (3.3-8.3) | <0.001 |
|  | 60-69 | 2.3 (1.4-3.9) | 0.001 | 1.9 (1.1-3.2) | 0.013 | 7.9 (5.1-12.4) | <0.001 | 6.2 (3.9-9.9) | <0.001 |
|  | >70 | 1.4 (0.7-2.9) | 0.332 | 1.2 (0.6-2.5) | 0.566 | 5.0 (2.7-9.3) | <0.001 | 3.8 (1.9-7.2) | <0.001 |
| Educational level | illiterates | 1 |  | 1 |  | 1 |  | 1 |  |
|  | Primary | 1.0 (0.7-1.3) | 0.817 | 1.1 (0.8-1.5) | 0.493 | 0.7 (0.5-0.9) | 0.022 | 0.7 (0.5-1.0) | 0.042 |
|  | Secondary | 0.5(0.4-0.8) | <0.001 | 0.8 (0.5-1.1) | 0.173 | 0.3 (0.2-0.5) | <0.001 | 0.4 (0.2-0.6) | <0.001 |
|  | Higher | 5 (0.3-1.9) | 0.019 | 0.6 (0.3-1.2) | 0.15 | 0.4 (0.2-0.7 | 0.04 | 0.4 (0.2-0.9) | 0.023 |
|  | other | 0.7(0.3-1.9) | 0.53 | 1.0 (0.4-2.7) | 0.929 | - | - | - |  |
|  | Unknown | - | - | - |  | 1.0 (0.6-1.6) | 0.869 | 1.2 (0.8-1.8) | 0.558 |
| Tattoo/acupuncture‡ | No | 1 |  | 1 |  | 1 |  | 1 |  |
|  | Yes | 1.6 (0.6-4.4) | 0.328 | 1.7 (0.6-4.6) | 0.309 | 1.2 (0.8-1.7) | 0.31 | 1.2 (0.8-1.8) | 0.451 |
| Traditional barber‡ | no | 1 |  | 1 |  | 1 |  | 1 |  |
|  | yes | 1.2 (0.9-1.6) | 0.138 | 1.1 (0.9-1.5) | 0.309 | 1.1 (0.9-1.5) | 0.414 | 1.4 (1.0-1.9) | 0.039 |
| Blood transfusion history‡ | No | 1 |  | 1 |  | 1 |  | 1 |  |
|  | Yes | 4.2 (2.6-6.9) | <0.001 | 2.9 (1.6-5.0) | <0.001 | 1.7 (1.1-2.5) | 0.09 | 1.0 (0.6-1.6) | 0.962 |
| Hospitalization history† | No | 1 |  | 1 |  | 1 |  | 1 |  |
|  | Yes | 2.0 (1.4-2.7) | <0.001 | 1.3 (0.9-1.8) | 0.195 | 1.8 (1.4-2.4) | <0.001 | 1.3 (0.9-1.8) | 0.214 |
| Invasive dental procedure‡ | No | 1 |  | 1 |  | 1 |  | 1 |  |
|  | Yes | 1.2 (0.7-2.0) | 0.586 | 0.8 (0.5-1.4) | 0.483 | 1.8 (1.4-2.4) | <0.001 | 1.0 (0.7-1.5) | 0.968 |
| Family history of hepatitis‡ | No | 1 |  | 1 |  | 1 |  | 1 |  |
|  | Yes | 2.5 (1.6-3.8) | <0.001 | 1.9 (1.2-3.0) | 0.005 | 3.1 (2.2 -4.4) | <0.001 | 3.0 (2.0-4.5) | <0.001 |
| HBV vaccination | No | 1 |  | 1 |  | 1 |  | 1 |  |
|  | Yes | 0.2 (0.1-0.8) | 0.023 | 0.2 (0.1-0.8) | 0.02 | 3.3 (1.9-5.6) | <0.001 | 2.6 (1.4-5.1) | 0.04 |
| Therapeutic injection† | No | 1 |  | 1 |  | 1 |  | 1 |  |
|  | Yes | 4.0 (2.6-6.2) | <0.001 | 3.3 (2.1-5.2) | 0 | 1.9 (1.4-2.6) | <0.001 | 1.6 (1.2-2.2) | 0.007 |
| Injection drug use | No | 1 |  | 1 |  | 1 |  | 1 |  |
|  | Yes | 1.7 (0.3-9.2) | 0.554 |  |  | 1.3 (0.3-4.7) | 0.717 |  |  |

†History in the last year

‡Ever history

*Among adults only

References

1. National Qualifications Framework of Pakistan In: Commission HE, Pakistan o, eds 2015.

2. District Goverment Dadu. <https://executivedistrictofficercdd.webnode.page/about-dadu/>.

3. Hyderabad District. 2010; <https://wiki.fibis.org/w/Hyderabad_District>.

4. Pakistan Emergency Situational Analysis - District Kashmore. 2014; <https://reliefweb.int/report/pakistan/pakistan-emergency-situational-analysis-district-kashmore-july-2014#:~:text=The%20district%20Kashmore%20was%20created,included%20in%20the%20district%20Kashmore>.

5. Mag P. Karachi Divided into Six Districts with Korangi New One. In:2013.

6. Qambar District. <https://www.wikiwand.com/simple/Qambar_District>.

7. Mansoor H. Thatta split to make Sujawal 28th district of Sindh. 2013; <https://www.dawn.com/news/1049252>.

# Figure legends

Supplementary Figure 1a. The overall prevalence of hepatitis B infection (HBsAg-positive) by districts of Sindh province across the 2007 and 2019 sero-survey.

Supplementary Figure 1b. The overall prevalence of hepatitis C exposure (HCV-Ab-positive) in districts of Sindh Province across the 2007 and 2019 sero-survey.
