# Supplementary material for: Changes in the prevalence of hepatitis B and C viral infections in Sindh province, Pakistan: Findings from two sero-surveys in 2007 and 2019
Source: J Viral Hepat. Author manuscript; Available in PMC 2024 Dec 23. (PMC7617255; doi:10.1111/jvh.13986)
Supplement: Supplementary file 2 [file EMS200023-supplement-Figure_S1_.docx]

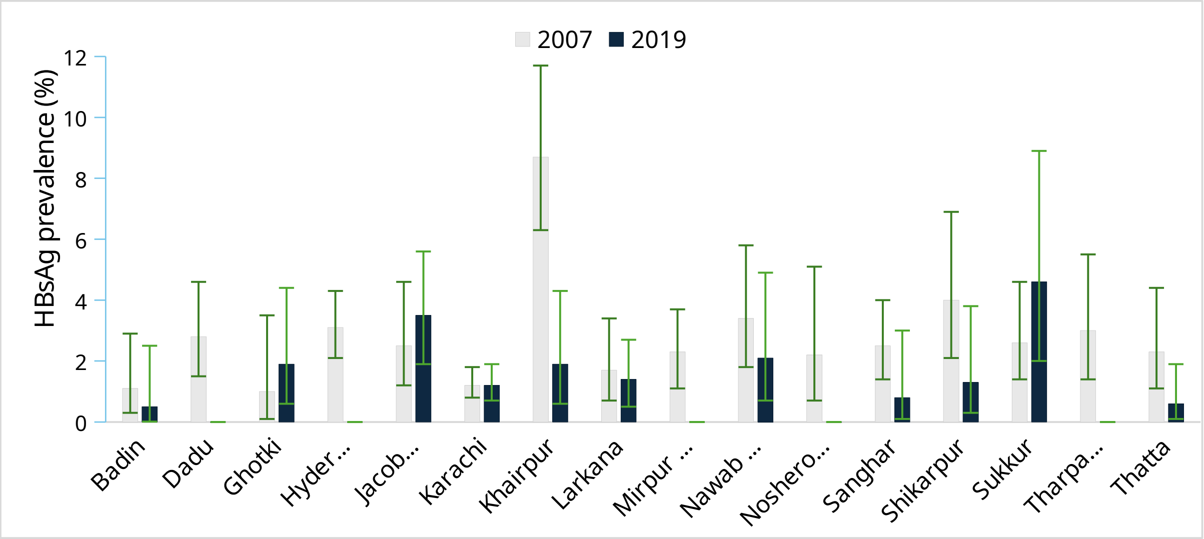
2007
2019
HBsAg prevalence (%)
12
10
8
6
4
2
0
Badin
Dadu
Ghotki
Hyder...
Jacob...
Karachi
Khairpur
Larkana
Mirpur ...
Nawab ...
Noshero...
Sanghar
Shikarpur
Sukkur
Tharpa...
Thatta
