# Supplementary material for: Changes in the prevalence of hepatitis B and C viral infections in Sindh province, Pakistan: Findings from two sero-surveys in 2007 and 2019
Source: J Viral Hepat. Author manuscript; Available in PMC 2024 Dec 23. (PMC7617255; doi:10.1111/jvh.13986)
Supplement: Supplementary file 3 [file EMS200023-supplement-Figure_S2_.docx]

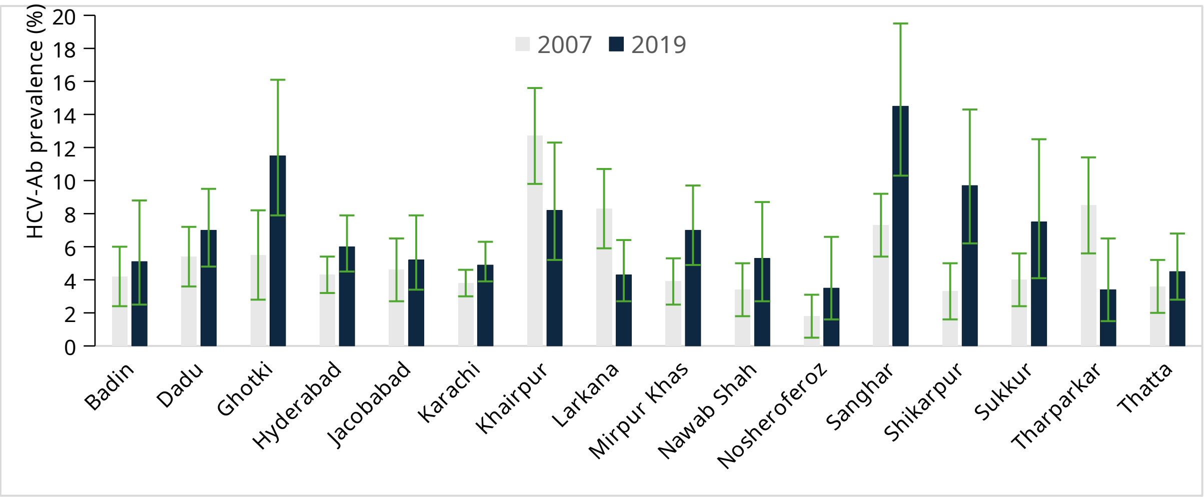
HCV-Ab prevalence (%)
0
2
4
6
8
10
12
14
16
18
20
2007
2019
Badin
Dadu
Ghotki
Hyderabad
Jacobabad
Karachi
Khairpur
Larkana
Mirpur Khas
Nawab Shah
Nosheroferoz
Sanghar
Shikarpur
Sukkur
Tharparkar
Thatta
